# Tele-neuropsychological Assessment of Children and Young People: A Systematic Review

**DOI:** 10.1007/s40817-023-00144-6

**Published:** 2023-05-31

**Authors:** Elise J. Walker, Fenella J. Kirkham, Hanne Stotesbury, Dagmara Dimitriou, Anna M. Hood

**Affiliations:** 1 Developmental Neurosciences Unit and Biomedical Research Centre, UCL Great Ormond Street Institute of Child Health, London, England; 2 Clinical and Experimental Sciences, University of Southampton, Southampton, England; 3 Sleep Education and Research Laboratory, Department of Psychology and Human Development, UCL Institute of Education, London, England; 4 Manchester Centre for Health Psychology, Division of Psychology and Mental Health, University of Manchester, Coupland 1 Building, Manchester, M15 6FH Manchester, England

**Keywords:** Tele-neuropsychology, Remote assessment, Pediatric, Cognition, Testing

## Abstract

The coronavirus pandemic identified a clinical need for pediatric tele-neuropsychology (TeleNP) assessment. However, due to limited research, clinicians have had little information to develop, adapt, or select reliable pediatric assessments for TeleNP. This preliminary systematic review aimed to examine the feasibility of pediatric TeleNP assessment alongside (1) patient/family acceptability, (2) reliability, and (3) the quality of the literature. Between May 2021 and November 2022, manual searches of PubMed, PsycINFO, and Google Scholar were conducted using terms related to “pediatric” and “tele-neuropsychology.” After extracting relevant papers with samples aged 0–22 years, predefined exclusion criteria were applied. Quality assessment was completed using the AXIS appraisal tool (91% rater-agreement). Twenty-one studies were included in the review, with reported qualitative and quantitative data on the feasibility, reliability, and acceptability extracted. Across included studies, TeleNP was completed via telephone/video conference with participants either at home, in a local setting accompanied by an assistant, or in a different room but in the same building as the assessor. Pediatric TeleNP was generally reported to be feasible (e.g., minimal behavioral differences) and acceptable (e.g., positive feedback). Nineteen studies conducted some statistical analyses to assess reliability. Most observed no significant difference between in-person and TeleNP for most cognitive domains (i.e., IQ), with a minority finding variable reliability for some tests (e.g., attention, speech, visuo-spatial). Limited reporting of sex-assigned birth, racialized identity, and ethnicity reduced the quality and generalizability of the literature. To aid clinical interpretations, studies should assess underexamined cognitive domains (e.g., processing speed) with larger, more inclusive samples.

**Supplementary Information:**

The online version contains supplementary material available at 10.1007/s40817-023-00144-6.

Pediatric neuropsychologists assess and support the complex needs of children and young people with brain-related disorders, illnesses, and injuries (e.g., an acquired brain injury, brain tumor, epilepsy, or neurodegenerative disease) (Fisher et al., 2020). Neuropsychological evaluations are targeted cognitive and behavioral assessments aimed at identifying a child’s relative strengths and weaknesses and generating recommendations for strategies and interventions to improve outcomes at home, school, and community engagement (Fisher et al., 2020; Hewitt et al., 2020).

Traditionally, neuropsychologists conduct assessments during in-person sessions, one-on-one in a quiet room, using manual or computerized tests (Tailby et al., 2020). This methodology enables rapport building, adapting test materials as needed, and careful behavioral observation (Barnett et al., 2018; Pritchard et al., 2020). However, more pediatric patients need assessment than the capacity of pediatric neuropsychological services. Additionally, pediatric patients in rural and socioeconomically deprived areas and from racialized communities face numerous structural inequities and barriers to services (Harder et al., 2020; Wright, 2020). The resultant pressure on pediatric neuropsychology services to support a larger and more diverse population has increased interest in remote neuropsychology assessment (Adjorlolo, 2015).

Remote neuropsychology assessment, where the assessor communicates with a patient from a different location using telephone or audio-visual technology, is commonly known as tele-neuropsychology (TeleNP) (Bilder et al., 2020; Stiers & Kewman, 1997). TeleNP has a short history, beginning with verbal working memory and intelligence (IQ) assessments using telephone calls in the late twentieth century (Cardon et al., 1992; Hodge et al., 2019a; Kent & Plomin, 1987). Despite the objective need, recent surveys of neuropsychologists indicate that TeleNP has been used infrequently in pediatric and adult clinical settings with limited data on clinical efficacy (Hammers et al., 2020). Indeed, until recently, there were no practice guidelines for pediatric TeleNP or adaptation of standardized measures for remote administration (Bilder et al., 2020). Restrictions on face-to-face interactions due to the SARS-CoV-2 (coronavirus) pandemic has seen interest in TeleNP accelerate quickly (Hammers et al., 2020), with many pediatric neuropsychology services forced to pivot to remote assessment (Zane et al., 2021). However, the evidence to support these rapid changes is limited.

Among the few research studies on TeleNP, most have recruited adult populations (Parsons et al., 2022). This is partly due to the ethical (e.g., safeguarding) and practical (e.g., knowledge constraints) considerations for pediatric assessments. For a comprehensive overview of ethical considerations for pediatric TeleNP, see Scott et al. (2022), Hewitt et al. (2020), and Bilder et al. (2020). Additionally, the core concerns for adult TeleNP may be compounded in pediatric evaluations, including access to and understanding of technology (Harder et al., 2020; Pritchard et al., 2020), privacy and security (Hewitt et al., 2020; Pritchard et al., 2020), and the importance of rapport building (Koterba et al., 2020). Neuropsychologists also report concerns about cognitive (e.g., ability to follow verbal instructions), behavioral (e.g., hyperactivity), and emotional (e.g., anxiety) difficulties that might prevent engagement and undermine the reliability of assessments (Koterba et al., 2020). Hewitt et al. (2020) reported concerns about school acceptance of pediatric TeleNP assessments and challenges about the role of caregivers who are often needed to guide the patient’s attention during assessments but whose presence may pose additional challenges (e.g., prompting).

Overall, there is strong interest but some hesitancy to implement pediatric TeleNP, with limited research to guide best practices. To date, only one previous review of pediatric tele-assessment has been conducted that only included speech and language assessments (Taylor et al., 2014). From the limited literature base, the authors identified five relevant studies. Of those, all except one had a sample size of less than 30, and there was high variability in participant characteristics and study methodologies.

The primary aim of this preliminary systematic review was to describe the current pediatric TeleNP research literature and examine the feasibility of pediatric TeleNP assessment. Secondary aims included considering (1) the reliability of pediatric TeleNP by extracting any available statistical comparisons of in-person versus TeleNP scores, (2) the acceptability of TeleNP through patient/family feedback, and (3) the generalizability and quality of the body of literature, including consideration of structural factors (i.e., racialized identity, geographic region).

## Methods

This systematic review constituted a narrative synthesis of the extracted data, followed the PRISMA (2020) guidance for systematic reviews (Page et al., 2021) (Online Resource 1) and was pre-registered on PROSPERO (CRD42021248969). Inclusion criteria were intentionally broad due to the limited research base of pediatric TeleNP. Included studies were peer-reviewed empirical articles (including clinical evaluations that were later published for research purposes) assessing TeleNP in clinical (e.g., young people with diagnosed or suspected special educational needs, disorders, or disabilities) and typically developing/non-clinical populations (e.g., no known or suspected special educational needs, disorders or disabilities). Our initial pre-registered age range was 3–18 years; however, to include four relevant studies, we extended the age range to 0–22 years to best capture TeleNP assessments for the pediatric population and account for the maturational period of the brain into early adulthood (Gogtay et al., 2004).

The review defines TeleNP as any neuropsychological assessment completed with the researcher not physically present during the assessment (i.e., video conferencing), including communicating with the participant by telephone. Any standardized neuropsychology assessment adapted for remote use was included, which refers to measures that examined cognitive and behavioral abilities through interviews, questionnaires, and testing (standardized or non-standardized). Studies which reported on non-neuropsychological assessment measures (i.e., audiology, sleep) were not assessed in the narrative synthesis. There were no restrictions on the publication date. Studies were excluded if no neuropsychological data were reported, no full text was available, it was not available in English, or it was conducted with an adult sample (i.e., over 22 years). Furthermore, this review does not contain data from technical reports or white papers available on commercial websites or published test manuals.

Between May 1, 2021, and November 30, 2022, the lead author (EJW) searched Google Scholar, PubMed, and PsycINFO (Online Resource 2). They combined three search strings: terms relating to “tele-assessment” and “video call,” terms relating to “pediatric,” and terms consisting of neuropsychology assessments (for example, “Delis- Kaplan Executive Function System” and “D-KEFS”). They examined the reference lists of each eligible study to identify further relevant work.

**Fig. 1:**
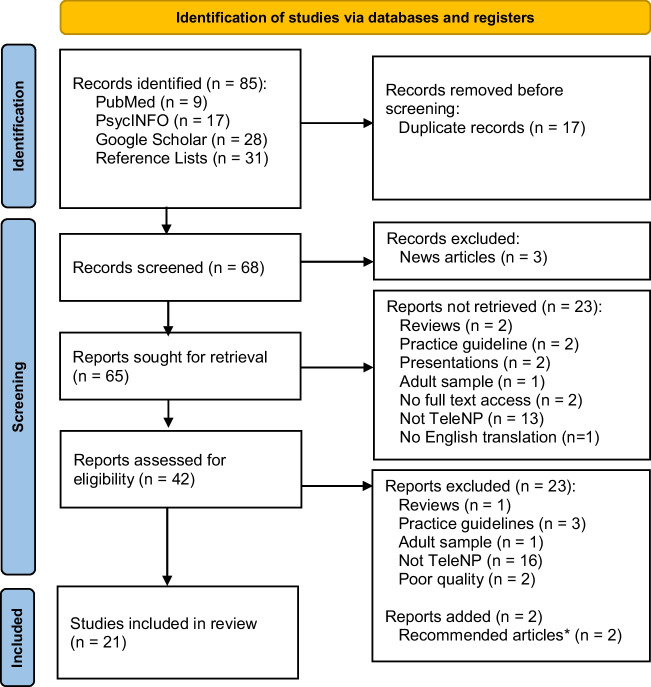
PRISMA flow diagram. *Articles recommended by author(s) of papers who were contacted for further sample details

Two authors (EJW and AMH) independently screened each identified paper for inclusion by first reading titles and abstracts, followed by full texts. Firstly, raw data, proportions [%], and means for participant demographic and clinical characteristics were extracted where available. Next, the names of TeleNP assessment measures included qualitative descriptions of TeleNP assessment administration (i.e., TeleNP behavioral observations), and quantitative TeleNP data (i.e., TeleNP raw and standardized scores, proportions [%] and means) were extracted. See our PRISMA flowchart of our search results (Fig. 1) (Page et al., 2021). Four studies were excluded as they did not have a standardized cognitive endpoint (i.e., functional behavioral analysis) (Barretto et al., 2006; Kovas et al., 2007; Machalicek et al., 2009; Wacker et al., 2013). Three studies did not report participant characteristic data (e.g., age, gender) and were contacted by email (Ciccia et al., 2011; Eriks-Brophy et al., 2008; Wright, 2020). Two authors replied, one providing the missing data (Ciccia et al., 2011) and the second unable to do so due to data deletion (Eriks-Brophy et al., 2008).

Reliability between in-person and TeleNP assessment scores was considered to be good if (a) papers reported no significant differences (*p* > 0.05) and (b) test–retest reliability (> 0.70) or correlation (> 0.50), or (c) interscorer agreement between in-person and TeleNP assessment (> 0.70). The Appraisal Tool for Cross-Sectional Studies (AXIS) assessed study quality (Downes et al., 2016). Initially, EJW and AMH coded all studies independently using AXIS. Each study was assigned a score from 0 to 19. Studies were considered to be of good quality if the score was greater than 70% (i.e., 14/19). Inter-rater reliability (κ statistic) was calculated by comparing the quality ratings between EJW and AMH.

## Results

Twenty-one studies were included in the final review (Fig. 1). Fifteen used a cross-sectional design (all participants completed TeleNP); four used a repeated measures design (participants completed TeleNP and in-person assessment); and two used a matched pairs design (one group completed TeleNP and another completed in-person assessment) (Table 1). All studies were peer-reviewed publications; three were conducted since the beginning of the coronavirus pandemic (i.e., since March 2020), and three recruited samples primarily for clinical evaluation. Three studies included participants aged under 3 years, of which Ransom et al. (2020) was based on a clinical interview, parent report, and play/behavioral observations; Ciccia et al. (2011) generated scores from parent report of emergent language; and Salinas et al. (2020) was based on a clinical practice model and did not report the full assessment measures used. Two studies (Hodge et al., 2019a, 2019b) used the same sample, but each study focused on a separate cognitive domain (i.e., language and IQ).

**Table 1:** Frequency table of study characteristics of the included studies

Study characteristics	Sample		Total no. of studies
	Clinical	Typically developing	
Total number of studies included	15	6	21 (100.00%)
Study year			
2000–2019	11	4	15 (71.43%)
> 2020	4	2	6 (28.57%)
Individual study sample size (N)			
< 30	9	0	9 (42.86%)
31–100	5	2	7 (33.33%)
100–999	1	3	4 (19.05%)
≥ 1,000	0	1	1 (4.76%)
Mean sample age (N)			
0–6	2	1	3 (14.28%)
7–10	5	3	8 (38.10%)
11–16	4	2	6 (28.57%)
17–22	0	0	0 (0.00%)
Not reported	4	0	4 (19.05%)
Design			
Repeated measures	2	1	3 (14.28%)
Cross-sectional (simultaneous scoring*)	10	0	10 (47.62%)
Cross-sectional (TeleNP scoring only)	2	2	4 (19.05%)
Matched pairs	1	3	4 (19.05%)
Country Location			
USA	6	5	11 (52.38%)
Canada	1	0	1 (4.76%)
Australia	8	0	8 (38.09%)
UK	0	1	1 (4.76%)
Population density			
City	0	1	1 (4.76%)
Rural	1	0	1 (4.76%)
Suburban	1	0	1 (4.76%)
Mixed	5	2	7 (33.33%)
Not reported	8	3	11 (52.38%)

Note. *Simultaneous scoring refers to an in-person researcher scoring the participant at the same time as another researcher scoring via video call. Clinical: young people with diagnosed or suspected special educational needs, disorders, or disabilities. Typically developing: no known or suspected special educational needs, disorders or disabilities

Four different methodologies were used in included studies, with two using more than one methodology in the same study: (1) the participant at home without an on-site facilitator and the off-site researcher (depending on the age of the participant) conducting the assessment via video or telephone call (*n* = 4); (2) the participant at home or local setting accompanied by an on-site facilitator (e.g., parent or university student) and the off-site researcher conducting testing via video or telephone call (*n* = 6); (3) the participant in a local setting accompanied by an on-site researcher, with the off-site researcher conducting testing via video or telephone call (both researchers simultaneously scoring) (*n* = 5); or (4) the participant in a different room but the same location as the researcher (e.g., hospital setting) either accompanied by a second researcher (both researchers simultaneously scoring *n* = 6) or a facilitator (e.g., university student; *n* = 2), with the primary researcher conducting testing via video or telephone call.

The 21 included studies reported 54 different assessment measures (Table 2). Most studies assessed multiple cognitive domains. All assessment measures were adapted from existing standardized measures. The most commonly researched measures for remote assessment were the Wechsler Intelligence Scale for Children–Fifth Edition (WISC-V) (Wechsler, 2014) (*n* = 5) to measure IQ and the Clinical Evaluation of Language Fundamentals–Fourth Edition (CELF-4) (Semel et al., 2006) (*n* = 4) to assess language. Only Ransom et al. (2020) included free-standing performance or symptom validity tests (PVT or SVT), and they did not report PVT or SVT results. Demographic characteristics extracted from included studies are reported in Table 3. After contacting authors for missing data, sex assigned at birth was missing for two studies. Ten studies did not report the primary language of participants, fifteen studies did not report the racialized identity of participants, and sixteen did not report the ethnic identity of participants (Table 3).

**Table 2:** Number of studies that assessed each cognitive domain

Cognitive domain	N of studies	Assessment measure(s) used	N of times measure was used
Intelligence (IQ)	8	Leiter International Performance Scale–Third Edition	1
		Stanford-Binet Intelligence Scale	1
		Wechsler Abbreviated Scale of Intelligence–Second Edition	2
		Wechsler Adult Intelligence Scale–Fourth Edition	1
		Wechsler Individual Achievement Test–Third Edition	1
		Wechsler Intelligence Scale for Children–Fifth Edition	5
		Wechsler Intelligence Scale for Children–Fourth Edition	1
Language	7	Bracken Basic Concept Scale: Expressive Form	1
		Clinical Evaluation of Language Fundamentals–Fourth Edition	4
		Comprehensive Test of Phonological Processing	1
		Expressive One Word Picture Vocabulary Test	1
		Preschool Language Scale–Fourth Edition	2
		Receptive-Expressive Emergent Language Test–Third Edition	1
		Screening Kit of Language and Development	1
		The Children’s Test of Nonword Repetition	1
Speech	3	Goldman-Fristoe Test of Articulation	1
		Oro-motor function*	2
		Single Word Articulation Test	1
		Speech sample – assessing intelligibility and fluency*	1
Literacy (i.e., reading, spelling)		Dalwood Spelling Test	1
	3	MultiLit (Sight Words & WordAttack)	1
		Neale Analysis of Reading Abilities–Third Edition	1
		Queensland University Inventory of Literacy	1
		South Australian Spelling Test	1
		Test of Word Reading Efficiency	1
		Test of Word Reading Efficiency–Second Edition	1
		Woodcock Reading Mastery Test–Third Edition	2
Executive Function	3	Behavior Rating Inventory of Executive Function	1
		Delis-Kaplan Executive Function System	2
Memory	2	Child and Adolescent Memory Profile	1
		California Verbal Learning Test–Children’s Version	1
		California Verbal Learning Test–Second Edition	1
		Memory Validity Profile	1
Adaptive Behavior	2	Adaptive Behavior Assessment System–Third Edition	1
		Vineland Adaptive Behavior Scale–Third Edition	2
Vocabulary	1	Peabody Picture Vocabulary Test–Third Edition	1
		Peabody Picture Vocabulary Test–Fourth Edition	1
Processing speed	1	Symbol Digit Modalities Test	1
Visual motor	1	Beery-Buktenica Developmental Test of Visual Motor Integration–Sixth Edition	1
Visual perceptual	1	Beery Visuo-Motor Index Test of Visual Perception	1
Multiple domains (i.e., assessment battery)	5	Learning/achievement	
		Differential Ability Scales–Second Edition	1
		Wide Range Assessment of Memory and Learning– Second Edition	2
		Woodcock-Johnson IV Tests of Achievement	2
	3	Diagnostic	
		Autism Diagnostic Interview–Revised	1
		Autism Diagnostic Observation Scale–Second Edition	1
		Autism Diagnostic Observation Schedule	1
		Childhood Autism Rating Scale–Second Edition	1
		Post Concussion Symptom Inventory–Second Edition	1
		The Modified Checklist for Autism in Toddlers	1
	2	Developmental	
		Bayley-III–Scales of Infant Development	1
		Developmental Profile–Third Edition	1
		NEPSY-II	2
	2	Cognitive	
		Woodcock-Johnson Tests of Cognitive Ability	2
	1	Behavioral	
		Behavior Assessment System for Children–Third Edition	1

*Note*: Many studies included assessments of multiple domains, so the *N* of studies does not equal to 21. *Qualitative description by the assessor

**Table 3:** Demographic characteristics of included studies

Demographic characteristic	N of studies where characteristic was reported (%) Total = 21	N of participants (%) Total = 7062
Sex assigned at birth		
Male	18	3266 (46.25%)
Female	18	3362 (47.61%)
Not reported	3	434 (6.15%)
Racialized identities*		
Black	6	66 (0.93%)
White	6	357 (5.06%)
Asian	5	17 (0.24%)
Biracial	1	1 (0.01%)
Native American	1	6 (0.08%)
Aboriginal Peoples**	1	7 (0.10%)
Pacific Islander	1	1 (0.01%)
Not classified	5	89 (1.26%)
Not reported	16	6518 (93.19%)
Ethnicity*		
Hispanic/Latinx	4	111 (1.57%)
Not classified	5	366 (5.18%)
Not reported	16	6585 (93.25%)
Primary language*		
English	10	5900 (83.55%)
Spanish/bilingual English and Spanish	2	22 (0.31%)
Not Classified	1	1 (0.01%)
Not reported	10	1139 (16.13%)

*Note.* Ethnicity has been defined in line with each countries use of the term. Two studies (Hodge et al., 2019a, 2019b) used the same sample, but did not report racialized identity, ethnicity, or primary language. *Some studies included participants from multiple racialized identities, ethnicities, and languages and as such, the total number of studies does not equal 21. **Included First Nations People, Métis, and Inuit peoples, though the number of participants in each group was not reported

### Quality

Two studies were excluded due to poor quality scores (i.e., limited reporting of participant characteristics and TeleNP methods (Cardon et al., 1992; Kent & Plomin, 1987). EW and AMH compared results at the data extraction stage with substantial agreement between the two raters (91.30%; Cohen’s κ = 0.62). Raters then obtained consensus on the two remaining studies. Of note, most studies (> 85%) did not provide justification for their sample size. See Online Resources 3, 4, and 5 for full results from the AXIS appraisal.

### Feasibility

See Table 4 for an overview of the characteristics of the included studies. Seven studies provided attrition rates (Dale et al., 2005; Harder et al., 2020; Ransom et al., 2020; Salinas et al., 2020; Sutherland et al., 2017, 2019; Worhach et al., 2021). Three studies reported that 2.86–27.37% of recruited participants were lost to follow-up or did not book a TeleNP appointment (Harder et al., 2020; Ransom et al., 2020; Salinas et al., 2020). Salinas et al. (2020) reported one participant did not attend their TeleNP appointment. Two studies had participants complete only partial TeleNP testing (Harder et al., 2020). Dale et al. (2005) reported missing/invalid TeleNP data of unspecified cause. Three studies reported that all participants completed all TeleNP testing (Salinas et al., 2020; Sutherland et al., 2017; Worhach et al., 2021).

**Table 4:** Summary of results for studies included in this systematic review

Authors (Year)	Method								Results
	*N* (Controls)	Sample	Age range (years)	Design	How assessed	Assessment location	Geographic location	Domains assessed	
Ciccia et al. (2011)	411 (10)	Mixed clinical volunteers	0–6	Cross-sectional and matched pairs	Video call	Local setting	USA	Speech; language	100% agreement between video call and in-person scores. Positive feedback
Dale et al. (2005)	5544	Twins cohort	6–7	Cross-sectional	Telephone call	At home	UK	Reading	Good agreement between telephone scores and teacher assessment (using a different measure)
Eriks-Brophy et al. (2008)	7	Speech and language difficulties	4–12	Cross-sectional	Video call	Local setting	Canada	Speech; language; Vocabulary	Good agreement between in-person and video call scores
Harder et al. (2020)	58	Demyelinating disorders	6–20	Repeated measures	Video call	At home	USA	Memory; processing speed; IQ; learning Battery; visuo-motor; visuo-perceptive; executive function	No significant differences between video call and in-person scores. Positive feedback
Hodge et al. (2019a)	37	Reading difficulties	8–11	Cross-sectional	Video call	Local setting	Australia	Reading; spelling	Good correlations between video call and in-person scores
Hodge et al. (2019b)	33	Reading difficulties	8–12	Cross-sectional	Video call	Local setting	Australia	IQ	Good correlations between video call and in-person scores. Positive feedback
Kronenberger et al. (2021)	28 (36)	Cochlear implants	9–22	Repeated measures	Video call	At home	USA	Cognitive and learning batteries; IQ; language; vocabulary	Some good correlations and no significant differences between video call and in-person scores. Positive feedback
Petrill et al. (2002)	52	Community volunteers	6–8	Matched pairs	Telephone call	At home	USA	IQ	Some good correlations between telephone call and in-person scores
Ragbeer et al. (2016)	3 (1)	Batten disease	10–16	Cross sectional	Video call	Same location, different room	USA	IQ; learning battery	Some good agreement between video call and in-person scores
Ransom et al. (2020)	105	Mixed clinical volunteers	1–21	Cross-sectional	Video call	At home	USA	Mixed batteries; IQ; memory; executive function; language; adaptive behavior	63% of participants were referred for face-to-face follow-up due to a lack of available assessments via TeleNP
Reese et al. (2013)	10 (11)	Developmental delay; autism	3–5	Cross-sectional	Video call	Same location, different room	USA	Autism diagnostic battery	Mostly good agreement between video conference and in-person scores. Positive feedback
Salinas et al. (2020)	67	Mixed clinical volunteers	2–18	Cross-sectional	Video call	At home	USA	Autism diagnostic battery; speech; adaptive behavior	100% able to complete all video call assessments
Sutherland et al. (2017)	23	Language impairment	8–12	Cross- sectional	Video call	Local setting	Australia	Language	Good inter-rater reliability and good agreement between video conference and in-person scores. No observed behavioral differences. Positive feedback
Sutherland et al. (2019)	13	ASD	9–12	Cross-sectional	Video call	Local setting	Australia	Language	Good correlations between video conference and in-person scores. No observed behavioral differences between conditions. Positive feedback
Waite et al. (2006)	6	Speech disorders	4–6	Cross-sectional	Video call	Same location, different room	Australia	Speech	Good agreement between video call and in-person scores
Waite et al. (2010a)	20	Reading/spelling difficulties	8–13	Cross-sectional	Video call	Same location, different room	Australia	Reading; spelling literacy	Mostly good agreement and good inter-rater reliability between video call and in-person scores
Waite et al. (2010b)	25	Language impairment	5–9	Cross-sectional	Video call	Same location, different room	Australia	Language	Good agreement, inter-rater reliability and correlations, and no significant differences between video conference and in-person scores
Waite et al. (2012)	20	Speech disorders	4–9	Cross-sectional	Video call	Same location, different room	Australia	Speech	Mostly good agreement and inter-rater reliability between video call and in-person scores
Worhach et al. (2021)	46	Sleep disorders	8–19	Repeated measures	Video call	At home	USA	IQ	No significant differences and mostly good correlations between video call and in-person scores. One subtest discontinued for poor correlations
Wright (2018)	120 (120)	Community volunteers	5–16	Matched pairs	Video call	Same location, different room	USA	Learning battery	No significant differences between video call and in-person scores
Wright (2020)	128 (128)	Community volunteers	6–16	Matched pairs	Video call	Same location, different room	USA	IQ	No significant differences between video conference and in-person scores except for one subtest

*Note*. This table does not report the results for non-neuropsychological assessments used in the included studies (i.e., audiology screening)

^*^Age range at first timepoint in a longitudinal study

Environmental and technical difficulties most commonly occurred within individual sessions than across sessions (Table 5). Harder et al. (2020) reported that 23% of individuals needed to borrow a study device. Ransom et al. (2020) found a significant correlation between device type (i.e., no laptop access) and TeleNP assessment attendance. Most studies that reported feasibility discussed technological difficulties or poor sound quality. For example, Hodge et al. (2019a) found that slow bandwidth and poor audio quality disrupted 6.06% sessions. However, according to assessor feedback, environmental distractions and technological difficulties were most often brief and did not appear to invalidate test performance or stop the assessment (Harder et al., 2020; Hodge et al., 2019b).

**Table 5:** Challenges experienced during tele-neuropsychology assessment—a summary of the major findings from included studies

Challenge	Examples
Environmental distractors	Background noise from a doorbell, phone ringing or family member/pet entering the room
Technological difficulties	
Camera setup	There was limited visibility of participant or assessment equipment
Desk setup	Desk was too high; headphones were the wrong size
Video quality	Screen froze; camera quality was poor; screen size was small
Audio quality	Sound produced echo, broke-up or there was a lag
Lighting	Participant’s face was shadowed or too bright
Slow bandwidth	Participant needed to refresh the computer or video call software
Device type	Participant was moving around more with a mobile device or tablet; device had a small screen
Participants behavior	
Attention/concentration	Participant became distracted
Fidgeting	Participant became fidgety, came off screen, or moved around a lot
Poor engagement	Participant would not complete an assessment task
Speaking too fast	Participant’s speech rate was too fast

### Acceptability

Acceptability was recorded via parent/carer/assessor/participant feedback using questionnaires (*n* = 8) (Ciccia et al., 2011; Harder et al., 2020; Hodge et al., 2019a, 2019b; Kronenberger et al., 2021; Reese et al., 2013) and the assessor’s behavioral observations of participants (*n* = 4) (Eriks-Brophy et al., 2008; Hodge et al., 2019a; Sutherland et al., 2017, 2019). All studies that used questionnaires found overall positive feedback. For example, Hodge et al. (2019a) reported that children found touchscreens “intuitive” during TeleNP, and caregivers observed positive behavioral responses. Sutherland et al. (2017) reported no assessor-observed behavioral differences between TeleNP and in-person assessment. When negative feedback was reported, it was generally related to audio or visual quality (Hodge et al., 2019a; Sutherland et al., 2019). Particularly, Kronenberger et al. (2021) found less acceptability for participants with cochlear implants, who reported more challenges from poor video-quality.

### Reliability

Nineteen of the included studies reported reliability statistics, of which six studies (assessing IQ, memory, and language) found good overall reliability per our predetermined criteria (i.e., no significant differences and good test–retest reliability, correlations, or interscorer agreement) (Kronenberger et al., 2021; Sutherland et al., 2017, 2019; Waite et al., 2010b; Worhach et al., 2021).

IQ was the most frequently researched domain. Seven studies provided reliability data for remote IQ assessments (Harder et al., 2020; Hodge et al., 2019a; Kronenberger et al., 2021; Petril et al., 2002; Ragbeer et al., 2016; Worhach et al., 2021; Wright, 2020). Four studies found no significant differences between most TeleNP and in-person IQ subtests and index scores, three reported good test–retest reliability, and one reported good inter-scorer agreement. However, four subtests from IQ batteries demonstrated either a significant difference (i.e., a processing speed subtest) (Wright, 2020), poor test–retest reliability (attention, short-term and verbal working memory subtests) assessed 1.6 years apart (Kronenberger et al., 2021), a poor correlation (i.e., perceptual reasoning subtest) (Worhach et al., 2021), or poor inter-scorer agreement (i.e., verbal fluency subtest) (Ragbeer et al., 2016) between TeleNP and in-person assessments.

Visuo-spatial abilities were assessed across five studies (including subtests within IQ assessments) (Harder et al., 2020; Hodge et al., 2019a; Ransom et al., 2020; Worhach et al., 2021; Wright, 2020), with four reporting reliability statistics. Of these, three studies reported no significant differences, and two reported some good correlations between TeleNP and in-person visuo-spatial assessment. In an interim analysis, Worhach et al. (2021) found poor reliability between in-person and TeleNP visuo-spatial assessment (one camera visible on the assessor’s screen), so switched WASI subtests (Matrix Reasoning used instead of Block Design), which increased reliability. Comparatively, Hodge et al. (2019a) successfully used the WISC-V Block Design subtest with two cameras split on the assessor’s screen. Wright (2020) used Q-Global’s adapted digital administration to reliably assess visuo-spatial abilities.

Five studies reported good interscorer agreement for language assessment, of which three also found good correlations, and two found no significant differences between in-person and TeleNP assessment (Ciccia et al., 2011; Eriks-Brophy et al., 2008; Sutherland et al., 2017, 2019; Waite et al., 2010b). Four studies reported on speech assessment reliability, all of which reported good interscorer agreement between in-person and TeleNP assessment—although there was variability in agreement for some individual oromotor variables (which requires interpretation of speech sounds) (Ciccia et al., 2011; Eriks-Brophy et al., 2008; Waite et al., 2006, 2012). Reading and literacy reliability were reported across four studies (Dale et al., 2005; Hodge et al., 2019b; Waite et al., 2010a; Wright, 2016, 2018), with good inter-rater agreement in two of these studies, no significant differences found by Wright (2018) and good correlations found in three of these studies) between TeleNP and in-person reading assessment.

Processing speed was assessed in five studies (three as part of IQ assessment) (Harder et al., 2020; Hodge et al., 2019a; Petril et al., 2002; Ransom et al., 2020; Wright, 2020), four of which reported reliability statistics. Two studies found good reliability; however, Wright, 2020 found a significant difference between in-person and TeleNP. Three studies reported on executive function assessments (Harder et al., 2020; Ransom et al., 2020; Salinas et al., 2020). Only Harder et al. (2020) reported reliability statistics, with no significant difference between in-person and TeleNP. Three studies reported on the reliability of memory assessment (Harder et al., 2020; Kronenberger et al., 2021; Ragbeer et al., 2016), of which Ragbeer et al. (2016) reported good interscorer agreement, Harder et al. (2020) and Kronenberger et al. (2021) reported no significant differences with some good test–retest reliability between in-person and TeleNP.

Diagnostic assessments for autism spectrum disorder were included in three studies (Ransom et al., 2020; Reese et al., 2013; Salinas et al., 2020). Only Reese et al. (2013) provided reliability statistics, finding no significant differences and high interscorer agreement overall. However, inter-rater agreement varied between items (15 items did not reach > 0.70 agreement), and one pointing subtest demonstrated a significant difference.

## Discussion

This preliminary systematic review examined the feasibility, acceptability, reliability, and quality of 21 published/peer-reviewed pediatric TeleNP studies. Some studies demonstrated significant differences or poorer correlations between TeleNP and in-person assessment subtests, but feasibility, reliability, and acceptability were most robust across IQ, memory, and language assessments. Across the included studies, feedback was generally positive, assessment completion rates were high, and there were mostly strong relationships between in-person and TeleNP assessment scores, particularly for studies including children three years and older. Barriers to TeleNP for assessors and participants (i.e., inadequate internet access) were not reported to have affected assessment completion. These findings align with research in adult populations (Parsons et al., 2022; Tailby et al., 2020). However, due to the small number of studies, evidence for the feasibility and acceptability of TeleNP for participants younger than 3 years old was limited.

The reliability of TeleNP varied most for speech, language, and reading comprehension assessments, which may have been due to audio and visual challenges. Differences in design (i.e., repeated measures versus simultaneous scoring), TeleNP setup (i.e., number of video-cameras), statistical analyses (i.e., intraclass correlations versus inter-rater reliability), and study periods (e.g., 1 week vs 1 year) made reliability across studies harder to interpret. Additionally, although recent work has shown that TeleNP assessing executive function and processing speed is reliable with adults (Parks et al., 2021), more studies are needed to determine reliability in the pediatric population.

Despite the primarily robust findings from the 21 TeleNP studies, generalizability is more challenging. There were very few large-scale TeleNP studies, with small samples (ns < 30) across large age ranges. Furthermore, most were pilot or feasibility studies, many were missing sample characteristics, the majority were conducted in the USA (*n* = 11; 52%) (Hammers et al., 2020), and there was little overlap in TeleNP assessments across studies. In addition, the most common type of TeleNP had two researchers (one in-person and one remote) simultaneous scoring. However, this research methodology does not best reflect how TeleNP would be used in clinical practice and may circumvent potential ethical challenges. Given that the majority of pediatric neuropsychologists do not embed free-standing PVT or SVT (Kirk et al., 2020) during in-person evaluations, it is perhaps unsurprising that they were utilized in only one of the TeleNP studies included in this review—the results of which were not reported (Ransom et al., 2020). Therefore, the failure or base rates of PVTs, which can be as high as 19% for in-person administrations, are unknown for pediatric TeleNP and must be incorporated in future research to inform formulation, interpretation, and recommendations (Kirk et al., 2020).

Our review indicates that the forced but often necessary shift to TeleNP since the beginning of the coronavirus pandemic in 2020 requires much more research to support this change. Significantly, the evidence base is not supported for specific pediatric assessment settings (e.g., adolescent forensic settings) where there are additional ethical (e.g., safeguarding) and practical (e.g., supervision) considerations. Privacy and informed consent would also need to be adapted for the specific risks (e.g., explaining to families the increased risk with electronic information transfer) for modified, remotely administered assessments (Scott et al., 2022).

Our review did not include any technical reports for remote assessment available on test publisher websites (e.g., PAR®) or in test manuals. This meant that some assessments with equivalency studies were missed. However, although these technical reports and white papers describe *how* remote administration should be completed, they do not yet include test scores that were normed specifically for TeleNP. Critically, tested developed specifically for TeleNP assessment (e.g., Reynolds Intellectual Assessment Scales–Second Edition; Wright, 2018) often cite equivalency studies based on smaller samples (e.g., ~ 100) compared to the larger normative samples (e.g., ~ 3000) used with in-person administrations, potentially reducing validity and reliability of modified assessment tools.

Children from under-resourced and marginalized communities were underrepresented in the included studies. This makes it difficult to conclude whether TeleNP is feasible or reliable for these populations, even though children from these communities are less likely to be able to attend in-person appointments (Lundine et al., 2020). Only one small study focused on a First Nation Indigenous population (Eriks-Brophy et al., 2008), and some studies excluded participants based on accessibility issues (e.g., hearing loss) (Waite et al., 2006). Future research should look to improve the inclusivity of samples to strengthen the evidence for TeleNP in clinical practice, which is particularly needed for families with limited or restricted transportation and those who live in rural communities (Adjorlolo, 2015).

This systematic review is preliminary as it includes a limited literature base of research primarily conducted before the coronavirus pandemic. We expect that there will be a larger number of studies in the next few years that can expand our knowledge of TeleNP. In our current study, having only one reviewer complete the initial screening may have reduced the number of eligible studies identified by up to 9% (Edwards et al., 2002). A meta-analysis was also beyond the scope of this research, as it would have been challenging to complete given most included studies used different cognitive assessments in TeleNP.

### Conclusion

Evidence from research studies indicates that pediatric TeleNP in clinical and non-clinical populations is feasible and acceptable. There is preliminary evidence for the reliability of some assessment measures. However, performance validity was not tested, and most studies included small, homogenous, mostly white samples with children over 3 years of age, limiting generalizability. Much more research with inclusive samples is needed before TeleNP can be used as an established and reliable option for clinical practice—particularly in specific complex populations such as pediatric forensic settings and for children with significant support needs. Tests used currently in TeleNP, even those modified for remote assessment, most often include normative samples tested during in-person evaluations. Thus, there is a critical need for tests specifically designed, tested, and normed for TeleNP. From such practice, appropriate guidance may be developed and further clinical pediatric TeleNP service models to be piloted.

## Supplementary Information

Below is the link to the electronic supplementary material.

Supplementary file1 (DOCX 57 KB)

## Data Availability

Data sharing is not applicable to this article as no new data were created or analyzed in this study.
