# Supplementary material for: Tele-neuropsychological Assessment of Children and Young People: A Systematic Review
Source: J Pediatr Neuropsychol. 2023 May 31:1–14. Online ahead of print. doi: 10.1007/s40817-023-00144-6 (PMC10231293; doi:10.1007/s40817-023-00144-6)
Supplement: Supplementary file 1 — Supplementary file1 (DOCX 57 KB) [file 40817_2023_144_MOESM1_ESM.docx]

**Online Resource 1.**

*PRISMA 2020 Checklist for Systematic Reviews*

| **Section and Topic** | **Item#** | **Checklist item** | **Location where item is reported** |
| --- | --- | --- | --- |
| **TITLE** | | |  |
| Title | 1 | Identify the report as a systematic review. | 1 |
| **ABSTRACT** | | |  |
| Abstract | 2 | See the PRISMA 2020 for Abstracts checklist. | 2 |
| **INTRODUCTION** | | |  |
| Rationale | 3 | Describe the rationale for the review in the context of existing knowledge. | 3, 4 |
| Objectives | 4 | Provide an explicit statement of the objective(s) or question(s) the review addresses. | 4 |
| **METHODS** | | |  |
| Eligibility criteria | 5 | Specify the inclusion and exclusion criteria for the review and how studies were grouped for the syntheses. | 5 |
| Information sources | 6 | Specify all databases, registers, websites, organisations, reference lists and other sources searched or consulted to identify studies. Specify the date when each source was last searched or consulted. | 5, 6, Figure 1 |
| Search strategy | 7 | Present the full search strategies for all databases, registers and websites, including any filters and limits used. | 5, 6, supplementary material |
| Selection process | 8 | Specify the methods used to decide whether a study met the inclusion criteria of the review, including how many reviewers screened each record and each report retrieved, whether they worked independently, and if applicable, details of automation tools used in the process. | 6 |
| Data collection process | 9 | Specify the methods used to collect data from reports, including how many reviewers collected data from each report, whether they worked independently, any processes for obtaining or confirming data from study investigators, and if applicable, details of automation tools used in the process. | 6 |
| Data items | 10a | List and define all outcomes for which data were sought. Specify whether all results that were compatible with each outcome domain in each study were sought (e.g. for all measures, time points, analyses), and if not, the methods used to decide which results to collect. | 6 |
|  | 10b | List and define all other variables for which data were sought (e.g. participant and intervention characteristics, funding sources). Describe any assumptions made about any missing or unclear information. | 6 |
| Study risk of bias assessment | 11 | Specify the methods used to assess risk of bias in the included studies, including details of the tool(s) used, how many reviewers assessed each study and whether they worked independently, and if applicable, details of automation tools used in the process. | N/A |
| Effect measures | 12 | Specify for each outcome the effect measure(s) (e.g. risk ratio, mean difference) used in the synthesis or presentation of results. | N/A |
| Synthesis methods | 13a | Describe the processes used to decide which studies were eligible for each synthesis (e.g. tabulating the study intervention characteristics and comparing against the planned groups for each synthesis (item #5)). | N/A |
|  | 13b | Describe any methods required to prepare the data for presentation or synthesis, such as handling of missing summary statistics, or data conversions. | 6 |
|  | 13c | Describe any methods used to tabulate or visually display results of individual studies and syntheses. | 5 |
|  | 13d | Describe any methods used to synthesize results and provide a rationale for the choice(s). If meta-analysis was performed, describe the model(s), method(s) to identify the presence and extent of statistical heterogeneity, and software package(s) used. | 6 |
|  | 13e | Describe any methods used to explore possible causes of heterogeneity among study results (e.g. subgroup analysis, meta-regression). | N/A |
|  | 13f | Describe any sensitivity analyses conducted to assess robustness of the synthesized results. | N/A |
| Reporting bias assessment | 14 | Describe any methods used to assess risk of bias due to missing results in a synthesis (arising from reporting biases). | N/A |
| Certainty assessment | 15 | Describe any methods used to assess certainty (or confidence) in the body of evidence for an outcome. | N/A |
| **RESULTS** | | |  |
| Study selection | 16a | Describe the results of the search and selection process, from the number of records identified in the search to the number of studies included in the review, ideally using a flow diagram. | 6, 7, Figure 1, supplementary material |
|  | 16b | Cite studies that might appear to meet the inclusion criteria, but which were excluded, and explain why they were excluded. | 6 |
| Study characteristics | 17 | Cite each included study and present its characteristics. | Table 4 |
| Risk of bias in studies | 18 | Present assessments of risk of bias for each included study. | N/A |
| Results of individual studies | 19 | For all outcomes, present, for each study: (a) summary statistics for each group (where appropriate) and (b) an effect estimate and its precision (e.g. confidence/credible interval), ideally using structured tables or plots. | (a) 8, 9, 10, 11  (b) N/A |
| Results of syntheses | 20a | For each synthesis, briefly summarise the characteristics and risk of bias among contributing studies. | Tables 1, 3 & 4 |
|  | 20b | Present results of all statistical syntheses conducted. If meta-analysis was done, present for each the summary estimate and its precision (e.g. confidence/credible interval) and measures of statistical heterogeneity. If comparing groups, describe the direction of the effect. | 8, 9, 10, 11 |
|  | 20c | Present results of all investigations of possible causes of heterogeneity among study results. | N/A |
|  | 20d | Present results of all sensitivity analyses conducted to assess the robustness of the synthesized results. | N/A |
| Reporting biases | 21 | Present assessments of risk of bias due to missing results (arising from reporting biases) for each synthesis assessed. | N/A |
| Certainty of evidence | 22 | Present assessments of certainty (or confidence) in the body of evidence for each outcome assessed. | N/A |
| **DISCUSSION** | | |  |
| Discussion | 23a | Provide a general interpretation of the results in the context of other evidence. | 11, 12 |
|  | 23b | Discuss any limitations of the evidence included in the review. | 12, 13 |
|  | 23c | Discuss any limitations of the review processes used. | 13 |
|  | 23d | Discuss implications of the results for practice, policy, and future research. | 12, 13 |
| **OTHER INFORMATION** | | |  |
| Registration and protocol | 24a | Provide registration information for the review, including register name and registration number, or state that the review was not registered. | 5 |
|  | 24b | Indicate where the review protocol can be accessed, or state that a protocol was not prepared. | 5 |
|  | 24c | Describe and explain any amendments to information provided at registration or in the protocol. | 5 |
| Support | 25 | Describe sources of financial or non-financial support for the review, and the role of the funders or sponsors in the review. | N/A |
| Competing interests | 26 | Declare any competing interests of review authors. | N/A |
| Availability of data, code and other materials | 27 | Report which of the following are publicly available and where they can be found: template data collection forms; data extracted from included studies; data used for all analyses; analytic code; any other materials used in the review. | N/A |

**Online Resource 2.**

*Summary of Search Terms used in PsychINFO*

| **Paediatric** | **TeleNeuropsychology** | **Cognitive Assessment** | **Cognitive Ability** |
| --- | --- | --- | --- |
| AB OR TI(   1. Child* 2. Infant* 3. Infancy 4. Pe?diatric* 5. Adolescent* 6. Teenager* 7. Youth 8. “Young people” 9. NOT adult 10. NOT geriatric   ) | AND (   1. Teleneuropsychology 2. Tele-neuropsych* 3. “Remote w/5 assessment*” 4. Tele-assessment 5. Teleassessment 6. Telehealth 7. Remote 8. Zoom 9. Videoconference 10. Videocall   ) | AND (   1. WISC 2. “Weschler W4 Intelligence N4 Scale*” 3. WISC-V 4. ABAS-3 5. "Adaptive Behaviour Assessment System" 6. "Child and Adolescent Memory Profile" 7. WASI 8. WASI-II 9. WPPSI 10. WPPSI-IV 11. Raven' 12. WIAT 13. WIAT-III 14. "Clinical evaluation of language fundamentals" 15. CELF-5 16. PLS 17. "Preschool Language scales" 18. "British Picture Vocabulary Scales" 19. PVS 20. "Renfrew Action Picture Test" 21. D-KEFS 22. "Delis-Kaplan Executive Function System" | OR (   1. Assessment 2. Intelligence 3. IQ 4. Memory 5. Attention 6. “Executive Function” 7. “Processing N3 Speed” 8. Language 9. Speech 10. Reading 11. Mathematics 12. Writing 13. “Nonverbal N3 intelligence”   ) |
|  |  | 1. “Trail Making" 2. Stroop 3. "Colo?r-Word Interference" 4. "Wisconsin Card Sorting Test" 5. WCST 6. "Children's Memory Scale" 7. CMS 8. "Rivermead behavioural memory test for children” 9. RBMT-C 10. TEA-Ch2 11. "Test of everyday attention for Children" 12. BADS 13. "Behavioural assessment of dysexecutive syndrome" 14. NEPSY-II 15. "Reynell Zinkin Scales" 16. ITVIC 17. "Intelligence scales for visually impaired children" 18. CBCL 19. "Child Behaviour Checklist” 20. "Leiter 3" 21. C-TONI 22. "TONI 4"   ) |  |

**Online Resource 3.**

*AXIS Appraisal Tool*

|  | **Question** | **Yes** | | **No** | **Don’t know** | **Comment** |
| --- | --- | --- | --- | --- | --- | --- |
| **Introduction** | | | | | |  |
| 1 | Were the aims/objectives of the study clear? | |  |  |  |  |
| **Methods** | | | | | |  |
| 2 | Was the study design appropriate for the stated aim(s)? | |  |  |  |  |
| 3 | Was the sample size justified? | |  |  |  |  |
| 4 | Was the target/reference population clearly defined? (Is it clear who the research was about?) | |  |  |  |  |
| 5 | Was the sample frame taken from an appropriate population base so that it closely represented the target/reference population under investigation? | |  |  |  |  |
| 6 | Was the selection process likely to select subjects/participants that were representative of the target/reference population under investigation? | |  |  |  |  |
| 7 | Were measures undertaken to address and categorise non-responders? | |  |  |  |  |
| 8 | Were the risk factor and outcome variables measured appropriate to the aims of the study? | |  |  |  |  |
| 9 | Were the risk factor and outcome variables measured correctly using instruments/measurements that had been trialled, piloted or published previously? | |  |  |  |  |
| 10 | Is it clear what was used to determined statistical significance and/or precision estimates? (e.g. p-values, confidence intervals) | |  |  |  |  |
| 11 | Were the methods (including statistical methods) sufficiently described to enable them to be repeated? | |  |  |  |  |
| **Results** | | | | | |  |
| 12 | Were the basic data adequately described? | |  |  |  |  |
| 13 | Does the response rate raise concerns about non-response bias? | |  |  |  |  |
| 14 | If appropriate, was information about non-responders described? | |  |  |  |  |
| 15 | Were the results internally consistent? | |  |  |  |  |
| 16 | Were the results presented for all the analyses described in the methods? | |  |  |  |  |
| **Discussion** | | | | | |  |
| 17 | Were the authors' discussions and conclusions justified by the results? | |  |  |  |  |
| 18 | Were the limitations of the study discussed? | |  |  |  |  |
| **Other** | | | | | |  |
| 19 | Were there any funding sources or conflicts of interest that may affect the authors’ interpretation of the results? | |  |  |  |  |
| 20 | Was ethical approval or consent of participants attained? | |  |  |  |  |
|  | **Total score** | |  |  |  |  |

**Online Resource 4**

*Quality Appraisals for Rater 1*

| **Paper** | **Questions** | | | | | | | | | | | | | | | | | | | | | | **Total** | **Risk of Bias** |
| --- | --- | --- | --- | --- | --- | --- | --- | --- | --- | --- | --- | --- | --- | --- | --- | --- | --- | --- | --- | --- | --- | --- | --- | --- |
|  | **1** | **2** | **3** | **4** | **5** | **6** | **7** | **8** | **9** | **10** | **11** | **12** | **13 & 14** | **15** | **16** | **17** | | **18** | | **19** | | **20** |  |  |
| Cardon et al., 1992 | 1 | 1 | 0 | 0 | 0 | 0 | 0 | 1 | 1 | 1 | 0 | 1 | 0 | 1 | 1 | 1 | | 0 | | 1 | | 0 | 9 | yes |
| Ciccia et al., 2011 | 1 | 1 | 0 | 0 | 1 | 1 | 0 | 1 | 1 | 0 | 1 | 1 | 1 | 1 | 0 | 1 | | 1 | | 1 | | 1 | 16 | no |
| Dale, et al., 2005 | 1 | 1 | 0 | 1 | 1 | 1 | 1 | 1 | 1 | 0 | 1 | 1 | 1 | 1 | 1 | 1 | | 0 | | 0 | | 0 | 14 | no |
| Eriks‐Brophy et al., 2008 | 1 | 1 | 0 | 1 | 1 | 1 | 0 | 1 | 1 | 1 | 1 | 1 | 0 | 1 | 1 | 0 | | 1 | | 1 | | 0 | 16 | no |
| Harder et al., 2020 | 1 | 1 | 0 | 1 | 1 | 1 | 1 | 1 | 1 | 1 | 1 | 1 | 1 | 1 | 1 | 1 | | 1 | | 1 | | 1 | 18 | no |
| Hodge et al., 2009 | 1 | 1 | 0 | 1 | 0 | 0 | 0 | 1 | 1 | 1 | 1 | 1 | 1 | 1 | 1 | 1 | | 1 | | 1 | | 1 | 14 | no |
| Hodge et al., 2019 | 1 | 1 | 0 | 1 | 1 | 1 | 0 | 1 | 1 | 1 | 1 | 1 | 1 | 1 | 1 | 1 | | 1 | | 0 | | 1 | 16 | no |
| Kent & Plomin, 1987 | 1 | 1 | 0 | 1 | 1 | 0 | 0 | 1 | 1 | 1 | 1 | 1 | 0 | 1 | 0 | 1 | | 0 | | 0 | | 0 | 11 | yes |
| Kovas, et al., 2007 | 1 | 1 | 1 | 1 | 1 | 1 | 0 | 1 | 1 | 1 | 1 | 1 | 0 | 1 | 1 | 1 | | 1 | | 1 | | 1 | 17 | no |
| Kronenberger, et al., 2021 | 1 | 1 | 0 | 1 | 1 | 0 | 0 | 1 | 1 | 1 | 1 | 1 | 1 | 1 | 1 | 1 | | 1 | | 1 | | 1 | 16 | no |
| Petrill et al., 2002 | 0 | 1 | 0 | 1 | 1 | 1 | 0 | 1 | 1 | 1 | 1 | 1 | 1 | 1 | 1 | 1 | | 0 | | 1 | | 1 | 16 | no |
| Ragbeer et al., 2006 | 1 | 1 | 0 | 1 | 1 | 1 | 0 | 1 | 1 | 1 | 1 | 1 | 0 | 0 | 1 | 1 | | 1 | | 0 | | 1 | 15 | no |
| Ransom et al., 2020 | 1 | 1 | 0 | 1 | 1 | 1 | 1 | 1 | 1 | 1 | 1 | 1 | 1 | 1 | 1 | 1 | | 1 | | 1 | | 1 | 19 | no |
| Reese et al., 2013 | 1 | 1 | 0 | 1 | 1 | 0 | 0 | 1 | 1 | 1 | 1 | 1 | 1 | 1 | 1 | 1 | | 1 | | 1 | | 0 | 14 | no |
| Salinas et. al., 2020 | 0 | 1 | 0 | 1 | 1 | 1 | 0 | 1 | 1 | 1 | 1 | 1 | 1 | 1 | 1 | 1 | | 1 | | 1 | | 0 | 14 | no |
| Sutherland et. al., 2017 | 1 | 1 | 1 | 0 | 1 | 1 | 0 | 1 | 1 | 1 | 1 | 1 | 1 | 1 | 1 | 1 | | 1 | | 1 | | 1 | 18 | no |
| Sutherland et al., 2019 | 1 | 1 | 0 | 1 | 1 | 0 | 0 | 1 | 1 | 1 | 1 | 1 | 1 | 1 | 1 | 1 | | 1 | | 1 | | 0 | 15 | no |
| Waite et al., 2006 | 1 | 1 | 0 | 1 | 1 | 0 | 0 | 1 | 1 | 1 | 1 | 1 | 1 | 1 | 1 | 1 | | 1 | | 0 | | 0 | 14 | no |
| Waite et al., 2010 | 1 | 1 | 0 | 1 | 1 | 0 | 0 | 1 | 1 | 1 | 1 | 1 | 1 | 1 | 1 | 1 | | 1 | | 1 | | 1 | 16 | no |
| Waite et al., 2010 | 1 | 1 | 0 | 1 | 1 | 0 | 0 | 1 | 1 | 1 | 1 | 1 | 1 | 1 | 1 | 1 | | 1 | | 1 | | 1 | 16 | no |
| Waite et al., 2012 | 1 | 1 | 0 | 1 | 1 | 0 | 0 | 1 | 1 | 1 | 1 | 1 | 1 | 1 | 1 | 1 | | 1 | | 1 | | 1 | 16 | no |
| Worhach et al., 2021 | 1 | 1 | 0 | 1 | 1 | 1 | 0 | 1 | 1 | 1 | 1 | 1 | 1 | 1 | 1 | 1 | | 1 | | 1 | | 0 | 17 | no |
| Wright 2016 | 1 | 1 | 1 | 1 | 1 | 1 | 0 | 1 | 1 | 1 | 1 | 1 | 1 | 1 | 1 | 1 | | 1 | | 1 | | 1 | 17 | no |
| Wright 2020 | 1 | 1 | 1 | 1 | 1 | 1 | 0 | 1 | 1 | 1 | 1 | 1 | 1 | 1 | 1 | | 1 | | 1 | | 1 | 1 | 18 | no |

**Online Resource 5**

*Quality Appraisals for Rater 2*

| **Paper** | **Questions** | | | | | | | | | | | | | | | | | | | **Total** | **Risk of Bias** |
| --- | --- | --- | --- | --- | --- | --- | --- | --- | --- | --- | --- | --- | --- | --- | --- | --- | --- | --- | --- | --- | --- |
|  | **1** | **2** | **3** | **4** | **5** | **6** | **7** | **8** | **9** | **10** | **11** | **12** | **13 & 14** | **15** | **16** | **17** | **18** | **19** | **20** |  |  |
| Cardon et al., 1992 | 1 | 1 | 0 | 0 | 0 | 0 | 0 | 1 | 1 | 1 | 1 | 1 | 0 | 1 | 1 | 0 | 0 | 0 | 0 | 9 | no |
| Ciccia et al., 2011 | 1 | 1 | 0 | 1 | 1 | 1 | 0 | 1 | 1 | 1 | 1 | 1 | 1 | 1 | 0 | 1 | 1 | 1 | 1 | 16 | yes |
| Dale, et al., 2005 | 0 | 1 | 0 | 1 | 1 | 1 | 1 | 1 | 1 | 0 | 1 | 1 | 1 | 1 | 0 | 1 | 1 | 0 | 1 | 14 | yes |
| Eriks‐Brophy et al., 2008 | 1 | 1 | 0 | 1 | 1 | 0 | 0 | 1 | 1 | 1 | 1 | 1 | 1 | 1 | 1 | 1 | 1 | 1 | 1 | 16 | yes |
| Harder et al., 2020 | 1 | 1 | 0 | 1 | 1 | 1 | 1 | 1 | 1 | 1 | 1 | 1 | 1 | 1 | 1 | 1 | 1 | 1 | 1 | 18 | yes |
| Hodge et al., 2009 | 1 | 1 | 0 | 1 | 1 | 0 | 0 | 1 | 1 | 0 | 1 | 1 | 1 | 1 | 1 | 1 | 1 | 0 | 1 | 14 | yes |
| Hodge et al., 2019 | 1 | 1 | 0 | 1 | 1 | 1 | 0 | 1 | 1 | 1 | 1 | 1 | 1 | 1 | 1 | 1 | 1 | 0 | 1 | 16 | yes |
| Kent & Plomin, 1987 | 1 | 1 | 0 | 1 | 1 | 0 | 0 | 1 | 1 | 0 | 1 | 1 | 1 | 1 | 0 | 1 | 0 | 0 | 0 | 11 | no |
| Kovas, et al., 2007 | 1 | 1 | 1 | 1 | 1 | 1 | 0 | 1 | 1 | 0 | 1 | 1 | 1 | 1 | 1 | 1 | 1 | 1 | 1 | 17 | yes |
| Kronenberger, et al., 2021 | 1 | 1 | 0 | 1 | 1 | 0 | 0 | 1 | 1 | 1 | 1 | 1 | 1 | 1 | 1 | 1 | 1 | 1 | 1 | 16 | yes |
| Petrill et al., 2002 | 1 | 1 | 0 | 1 | 1 | 1 | 0 | 1 | 1 | 1 | 1 | 1 | 1 | 1 | 1 | 1 | 0 | 1 | 1 | 16 | yes |
| Ragbeer et al., 2006 | 1 | 1 | 1 | 1 | 1 | 1 | 0 | 1 | 1 | 1 | 1 | 1 | 0 | 1 | 1 | 1 | 1 | 0 | 1 | 15 | yes |
| Ransom et al., 2020 | 1 | 1 | 1 | 1 | 1 | 1 | 1 | 1 | 1 | 1 | 1 | 1 | 1 | 1 | 1 | 1 | 1 | 1 | 1 | 19 | yes |
| Reese et al., 2013 | 1 | 1 | 0 | 1 | 1 | 0 | 0 | 1 | 1 | 0 | 1 | 1 | 1 | 1 | 1 | 1 | 1 | 1 | 0 | 14 | yes |
| Salinas et. al., 2020 | 0 | 1 | 0 | 0 | 1 | 1 | 0 | 1 | 1 | 0 | 1 | 1 | 1 | 1 | 1 | 1 | 1 | 1 | 0 | 14 | yes |
| Sutherland et. al., 2017 | 1 | 1 | 1 | 1 | 1 | 1 | 0 | 1 | 1 | 1 | 1 | 1 | 1 | 1 | 1 | 1 | 1 | 1 | 1 | 18 | yes |
| Sutherland et al., 2019 | 1 | 1 | 0 | 1 | 1 | 0 | 0 | 1 | 1 | 1 | 1 | 1 | 1 | 1 | 1 | 1 | 1 | 1 | 0 | 15 | yes |
| Waite et al., 2006 | 1 | 1 | 0 | 1 | 1 | 0 | 0 | 1 | 1 | 1 | 1 | 1 | 1 | 1 | 1 | 1 | 1 | 0 | 0 | 14 | yes |
| Waite et al., 2010 | 1 | 1 | 0 | 1 | 1 | 0 | 0 | 1 | 1 | 1 | 1 | 1 | 1 | 1 | 1 | 1 | 1 | 1 | 1 | 16 | yes |
| Waite et al., 2010 | 1 | 1 | 0 | 1 | 1 | 0 | 0 | 1 | 1 | 1 | 1 | 1 | 1 | 1 | 1 | 1 | 1 | 1 | 1 | 16 | yes |
| Waite et al., 2012 | 1 | 1 | 0 | 1 | 1 | 0 | 0 | 1 | 1 | 1 | 1 | 1 | 1 | 1 | 1 | 1 | 1 | 1 | 1 | 16 | yes |
| Worhach et al., 2021 | 1 | 1 | 0 | 1 | 1 | 1 | 0 | 1 | 1 | 1 | 1 | 1 | 1 | 1 | 1 | 1 | 1 | 1 | 1 | 17 | yes |
| Wright 2016 | 1 | 1 | 1 | 0 | 1 | 1 | 1 | 1 | 1 | 1 | 1 | 1 | 1 | 1 | 1 | 1 | 1 | 1 | 0 | 17 | yes |
| Wright 2020 | 1 | 1 | 1 | 1 | 1 | 1 | 0 | 1 | 1 | 1 | 1 | 1 | 1 | 1 | 1 | 1 | 1 | 1 | 1 | 18 | yes |
